# Non-invasive prenatal testing for autosomal recessive disorders: A new promising approach

**DOI:** 10.3389/fgene.2022.1047474

**Published:** 2022-11-03

**Authors:** Yusra Alyafee, Abeer Al Tuwaijri, Muhammad Umair, Mashael Alharbi, Shahad Haddad, Maryam Ballow, Latifah Alayyar, Qamre Alam, Saleh Althenayyan, Nadia Al Ghilan, Aziza Al Khaldi, Majid S. Faden, Hamad Al Sufyan, Majid Alfadhel

**Affiliations:** ^1^ Medical Genomics Research Department, King Abdullah International Medical Research Center (KAIMRC), King Saud Bin Abdulaziz University for Health Sciences, King Abdulaziz Medical City, Ministry of National Guard Health Affairs, Riyadh, Saudi Arabia; ^2^ Clinical Laboratory Sciences Department, College of Applied Medical Sciences, King Saud Bin Abdulaziz University for Health Sciences (KSAU-HS), Riyadh, Saudi Arabia; ^3^ Maternal Fetal Medicine Department, King Abdulaziz Medical City, Ministry of National Guard Health Affairs, Riyadh, Saudi Arabia; ^4^ Department of Pathology and Laboratory Medicine, King Abdulaziz Medical City, Ministry of National Guard Health Affairs, Riyadh, Saudi Arabia; ^5^ Department of Obstetrics and Gynaecology, Maternal Fetal Medicine, King Fahad Medical City, Riyadh, Saudi Arabia; ^6^ Assisted Reproductive Technology Laboratories, Thuriah Medical Center, Riyadh, Saudi Arabia; ^7^ Genetics and Precision Medicine Department (GPM), King Abdullah Specialized Children’s Hospital, King Saud Bin Abdulaziz University for Health Sciences, King Abdulaziz Medical City, MNG-HA, Riyadh, Saudi Arabia

**Keywords:** autosomal recessive, AR, fetal DNA, non-invasive prenatal testing for monogenic disorders (NIPTM), Single gene, whole-genome amplification

## Abstract

**Background:** In pregnant women at risk of autosomal recessive (AR) disorders, prenatal diagnosis of AR disorders primarily involves invasive procedures, such as chorionic villus sampling and amniocentesis.

**Methods:** We collected blood samples from four pregnant women in their first trimester who presented a risk of having a child with an AR disorder. Cell-free DNA (cfDNA) was extracted, amplified, and double-purified to reduce maternal DNA interference. Additionally, whole-genome amplification was performed for traces of residual purified cfDNA for utilization in subsequent applications.

**Results:** Based on our findings, we detected the fetal status with the family corresponding different genes, i.e., *LZTR1*, *DVL2*, *HBB*, *RNASEH2B*, and *MYO7A*, as homozygous affected, wild-type, and heterozygous carriers, respectively. Results were subsequently confirmed by prenatal amniocentesis. The results of AmpFLSTR™ Identifiler™ presented a distinct profile from the corresponding mother profile, thereby corroborating the result reflecting the genetic material of the fetus.

**Conclusion:** Herein, we detected AR disease mutations in the first trimester of pregnancy while surmounting limitations associated with maternal genetic material interference. Importantly, such detection strategies would allow the screening of pregnant women for common AR diseases, especially in highly consanguineous marriage populations. This technique would open avenues for the early detection and prevention of recessive diseases among the population.

## 1 Introduction

The discovery of cell-free fetal DNA (cffDNA) in the mother’s plasma by Lo et al. (1997) in 1997 has paved the way for developing new applications in clinical practice that rely on analyzing fetal genetic material (Rather et al., 2019). cffDNA comprises approximately 3%–13% of the mother’s cell-free DNA (cfDNA) and is released into maternal circulation from placental cells undergoing apoptosis. The amount of cffDNA increases with gestational age and is cleared hours after delivery (Taglauer et al., 2014). Rapid advances in sequencing technology have facilitated the analysis of circulating cffDNA with considerable sensitivity and specificity. Since 2011, cffDNA has been employed to screen pregnant women at high risk for common fetal trisomies (Benachi et al., 2015).

Currently, the use of cffDNA for fetal screening is a rapidly developing technology, but its application remains limited. These limitations are primarily attributed to the overwhelming quantity of maternal genetic material when compared with that of fetal origin (Gerson and O'Brien, 2018). With the resolution of technical challenges and advances in genetic research, the use of cffDNA for fetal screening is expected to grow (Dhallan et al., 2007). In 2011, the UK National Health Service applied non-invasive prenatal testing (NIPT) in clinical practice for fetal sex determination (Hill et al., 2011). In 2014, the prediction of the fetal RhD type was introduced into clinical practice for the early detection and management of pregnancies in RhD-immunized women in Europe (Clausen, 2014). Cell-free fetal sex determination is currently used in NIPT services for several families with X-linked disorders, such as Duchenne muscular dystrophy and hemophilia. NIPT has also been introduced in the UK for *de novo* or paternal autosomal dominant mutations, such as achondroplasia. The main approach for NIPT tests is PCR, followed by digestion with a restriction enzyme (PCR-RED) (Chitty et al., 2011). The development of a new expanded next-generation sequencing panel has allowed the accurate assessment of several variants, as previously described for autosomal dominant disorders (Zhang et al., 2019). Meanwhile, identifying the most accurate, sensitive, and specific approach for NIPD of autosomal recessive (AR) disorders, in which both parents carry the same mutation as the fetus, presents considerable challenges. This can be attributed to the low level of fetal mutant alleles when compared with the high background of the maternal mutant allele in the circulating cfDNA in maternal plasma. In NIPT for AR disorders, the non-invasive droplet-digital PCR (ddPCR) assay requires the design of specific probes for familial mutations (Chang et al., 2018). Additional alternative approaches have been introduced, such as NIPT for β-thalassemia, in which haplotype dosage is assessed using semiconductor sequencing and target capture enrichment combined with specific target capture sequences (Saba et al., 2017; Koumbaris et al., 2019). These techniques have allowed the identification of haplotypes inherited by the fetus. Unfortunately, the demographic characteristics of examined samples were not provided, which is essential for detecting positivity rates. Therefore, additional improvements remain critical.

For utilizing cffDNA in AR disease screening, technological advances are particularly important in populations with high consanguineous marriages, where rare AR disorders are common (Alkuraya, 2014). Developing a screening strategy to identify family-specific AR disorders during the early stages of pregnancy remains crucial.

In the present study, we developed and validated a highly sensitive and specific whole-genome amplification approach for cffDNA purification and enrichment to screen pregnant women at high risk of having a child with family-specific AR disease. Our approach could overcome limitations such as maternal genomic interference and low fetal fractions. Furthermore, we demonstrated the feasibility of applying this strategy to pregnant women in the first trimester. The developed method could facilitate substantial advances in detecting and preventing several family-oriented AR diseases, especially in highly consanguineous marriage populations.

## 2 Materials and methods

### 2.1 Study approval and consent

This study was approved by the Institutional Review Board (IRB) of the King Abdullah International Medical Research Center (KAIMRC), Riyadh, Saudi Arabia (NRC21R/083/03 approved 1 September 2022). The patients underwent a full clinical assessment for genetic and rare diseases at the Obstetrics and Gynecology Clinic of the National Guard Hospital (NGH), Riyadh, Saudi Arabia. Written informed consent for conducting the experiments and subsequent publication and storage of clinical data was provided and signed by each pregnant woman enrolled in this study.

### 2.2 Study subjects

We recruited four pregnant women and partners who were carriers of an AR disorder. All women had conceived naturally and had a singleton pregnancy with a gestational age of >10 weeks. Full and detailed family histories of any genetic disorder were obtained, along with the weight and height of each patient. An ultrasound scan was performed to confirm the number of fetuses and gestational age.

### 2.3 Samples collection, genomic DNA extraction, and DNA library preparation

Approximately 10 ml of the peripheral venous blood sample was collected in an EDTA tube from each pregnant woman, and each tube was labeled with a unique medical record number and accession number. Extraction and library amplification of cffDNA were performed as described earlier (Alyafee et al., 2021a). Moreover, 15 ml of amniotic fluid was collected, and fetus-precipitated cells underwent direct DNA extraction using Gentra^®^ Puregene^®^ Tissue (Hilden, Germany) in accordance with the manufacturer’s protocol.

### 2.4 cffDNA library purification and size selection

cffDNA libraries were size selected to ensure that fragments were within the suitable read length range of <160 bp length. First, paramagnetic beads were added to the DNA library pool, binding to the larger unwanted fragments in the sample, while the supernatant containing the desired cffDNA library was retained. Furthermore, the size-selected DNA library pool was quantified in accordance with the manufacturer’s protocol (High Sensitivity DNA Reagents & Agilent 2,100 Bioanalyzer) (Liao et al., 2014). Subsequently, the cffDNA library product was purified and cleaned twice using magnetic beads (G-Biosciences) to substantially eliminate larger DNA of maternal origin and increase the cfDNA concentration of fetal origin (Sparks et al., 2012).

### 2.5 Whole-genome amplification for cffDNA

The cffDNA enrichment was performed using a ReproSeq kit (Cat No. A34899, Thermo Fisher Scientific) according to the manufacturer’s protocol.

### 2.6 Detection of direct point mutation detection and cfDNA of maternal origin

Whole-genome amplification (WGA) products of cffDNA were used to detect direct mutations using Sanger sequencing combined with short tandem repeat (STR) identifier analysis for a haplotyping-based approach (Asiri et al., 2020; Umair et al., 2020). For regions flanking mutations in *DVL2*, *LZTR1*, *HBB*, *MYO7A*, and *RNASEH2B* genes, the PCR reactions were performed using cffDNA WGA products in 20 μl reaction mixture consisting of 1× PCR buffer, 5 μl of 1/10 μl of the MDA product or genomic DNA, 0.5 pmol/μl of each primer set, and 10 μl AmpliTaq 360 Gold DNA Polymerases (Applied Biosystems). The initial enzyme activation and denaturation were performed at 95°C for 10 min. Thermal cycling was performed under the following conditions: 30 cycles of denaturation at 94°C for 1 min, annealing at 55°C for 1 min, and extension at 70°C for 1 min. The PCR products were subjected to cycle sequencing using a BigDye Terminator version 3.1 Ready Reaction Cycle Sequencing Kit with the forward primer. The amplified products were loaded onto an ABI Prism 3710XL Genetic Analyzer with the POP- 7 polymer (Barhoumi et al., 2019; Alhamoudi et al., 2020).

### 2.7 Haplotype analysis and detection of maternal cfDNA contamination

A panel of informative STR markers is located within a 2 MB interval around *LZTR1*, *DVL2*, and *HBB*. Linked markers were selected using the UCSC Genome Browser (http://genome.ucsc.edu/). The residual WGA product of cffDNA was used to confirm the haplotyping results. All forward primers were fluorescently labeled at the 5′ end by either fluorescein (Fam) or hexachlorofluorescein (HEX). The PCR was performed in 20 μl reactions, containing 5 μl of WGA product of cffDNA or 50 ng/μl genomic DNA, 10 µl of AmpliTaq 360 Gold DNA Polymerases (Applied Biosystems), and 0.5 pmol/μl of each primer set. Initial enzyme activation and denaturation were performed at 95°C for 10 min, followed by thermal cycling under the following conditions: denaturation at 94°C for 1 min, annealing at 50°C for 1 min, and extension at 70°C for 1 min for 25 cycles of amplification. Fragment analysis was performed using GeneScan software and ABI PRISM 3710 Genetic Analyzer (Applied Biosystems). Finally, to detect cfDNA of maternal origin, AmpFLSTR™ Identifiler™ Plus PCR Amplification kit was used as described by the manufacturer (Applied Biosystems).

## 3 Results

In the present study, we recruited four pregnant women to detect fetal-related AR disorders in the maternal blood using cfDNA. All eligible participants were of Saudi Arabian ethnicity, single fetuses, and carriers with partners for at least one AR disorder and at high risk of having an affected child. The average age of the recruited pregnant women was (33.8 ± 5.6) years (range, 26–39 years), and the average gestational age at the time of NIPT was 12 weeks (range, 10–14 weeks). The average cffDNA pre-enrichment fraction of reported cases was 9%, ranging from 6 to 13%. Clinical descriptions of all pregnant women recruited are summarized in Table 1.

**TABLE 1:** General description for recruited patients.

Case number	Fetal fraction%	Maternal age (years)	Gestational age (weeks)
CASE1	9	38	13
CASE1	9	38	13
CASE2	6	39	11
CASE3	8	26	10
CASE4	13	28	14

**FIGURE 1:**
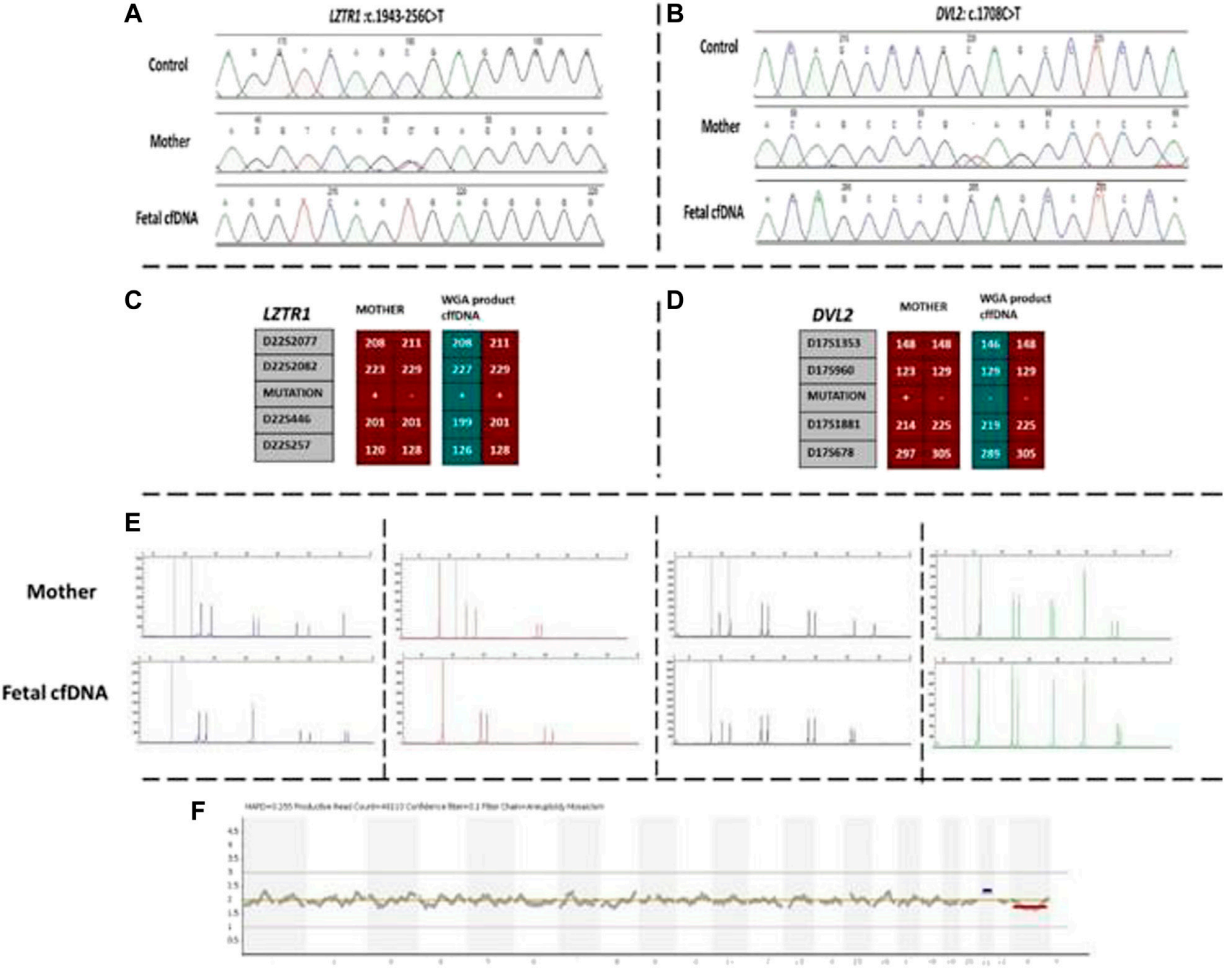
Findings of case 1. **(A)** Sanger results for *LZTR1* gene for mother and WGA product of cffDNA. **(B)** Sanger results for *DVL2* gene for mother and WGA product of cffDNA. **(C,D)** Karyomaps comparison for *LZTR1* and *DVL2*, respectively. **(E)** AmpFLSTR™ Identifiler™ Plus kit results for the mother and WGA product of cffDNA to detect maternal interference. **(F)** Ion reporter genome viewer for the WGA product of the cffDNA showing the high risk of trisomy 21 in the male fetus. cffDNA, cell-free fetal DNA; WGA, Whole-genome amplification.

Our first case was a 38-year-old pregnant woman with a previous history of an affected daughter with two pathogenic homozygous variants in [*DVL2* (NM_004422.3): c.1690C>T; (p.Gln564Ter) and *LZTR1* (NM_006767.4): c.1943–256C>T] genes. The patient was recruited for subsequent pregnancy at 13 weeks of gestation after ultrasound imaging revealed fetal hydrops. Regular NIPT results showed a high risk of Down syndrome, which was confirmed by arrayCGH. Moreover, the WGA product of cffDNA was used to detect whether the fetus was affected by the two previously mentioned mutated genes. Based on the direct point mutation detection results for both genes, the fetus was homozygous for the *LZTR1* gene and wild-type for the *DVL2* gene. To confirm the accuracy of obtained results, informative STR linking analysis was performed for both genes to detect the presence of the paternal allele, as well as exclude allele dropout and maternal genetic material interference. Moreover, the AmpFLSTR kit results revealed external and maternal contamination. The results of this patient are summarized in Figure 1.

**FIGURE 2:**
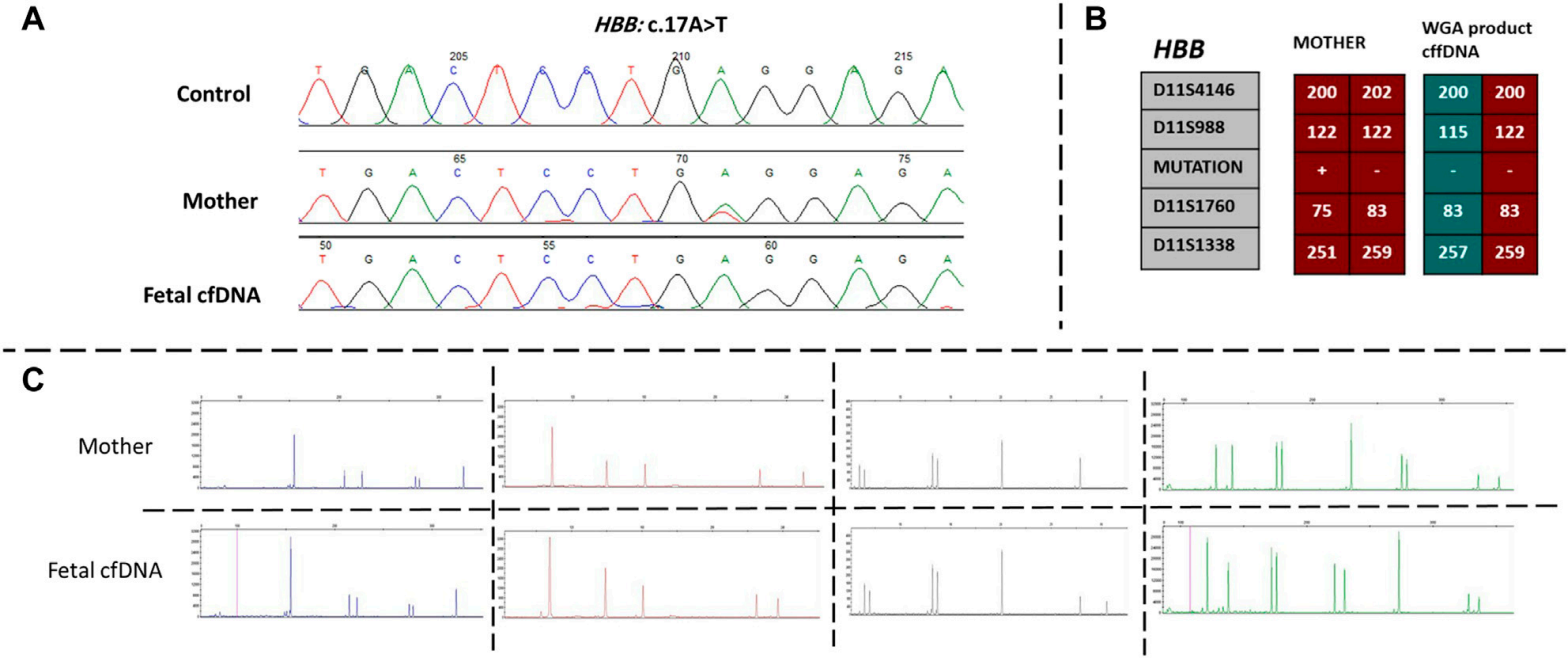
Findings of case 2. **(A)** Sanger results for *HBB* gene for mother and WGA product of cffDNA. **(B)** Karyomaps comparison for *HBB*, gene between the mother and WGA product of cffDNA to detect the paternal allele and exclude ADO probability. **(C)** AmpFLSTR™ Identifiler™ Plus kit results for the mother and WGA product of cffDNA to detect maternal interference. ADO, allele dropout; cffDNA, cell-free fetal DNA; WGA, Whole-genome amplification.

**FIGURE 3:**
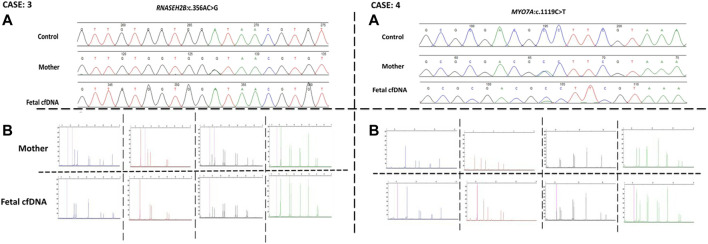
Case 3 and case 4 findings. **(A)** Sanger results for *RNASEH2B,* and *MYOA7A* genes respectively, for the mothers and WGA products of cffDNA. **(B)** AmpFLSTR™ Identifiler™ Plus kit results for the mothers and WGA product of cffDNA to detect maternal interference.

Next, we recruited additional patients to confirm that the developed method could be applied to different diseases and genomic regions. As described in Table 2, we enrolled three additional families with distinct genes involving different chromosomes. Results of direct point mutation of all genes using WGA products of cffDNA revealed the wild-type status of the fetus for cases 2 [*HBB* (NM_000518.5):c.17C>T; (p.Pro6Leu)] and 4 [*MY O 7A* (NM_001127180.2):c.1190C>T; (p.Ala397Val)], whereas the fetus of case 3 exhibited a carrier status [*RNASEH2B* (NM_024570.4):c.356A>G; (p.Asp119Gly)]. Similar to the first patient, allele dropout and maternal cell contamination interference were excluded by several informative STRs around related genes, and prenatal genetic amniocenteses confirmed the cffDNA results. The detailed results for all recruited patients are presented in Figures 2, 3.

**TABLE 2:** Summary of results and outcomes observed.

Case number	Gene	Variant	Mother status	WGA product cffDNA	Confirmation	Maternal genomic DNA interferance
CASE1	LZTR1	c.1943–256C>T	−/+	+	+	NO
CASE1	DVL2	c.1690C>T	−/+	−/−	−/−	NO
CASE2	HBB	c.17A>T	−/+	−	−/−	NO
CASE3	MYO7A	c.1190C>T	−/+	−/+	−/+	NO
CASE4	RNASEH2B	c.356A>G	−/+	−	−/−	NO

## 4 Discussion

Following the discovery of cffDNA by Lo et al. (1997) in pregnant women, NIPT with high-throughput sequencing has been widely adopted for the clinical detection of chromosomal abnormalities. This high-throughput DNA sequencing technique can rapidly and effectively detect large-scale genetic alterations with high accuracy and specificity for trisomies T21, T18, and T13 (Gregg et al., 2016).

Currently, NIPT for aneuploidy is commonly used worldwide as a preliminary screening test for the most common fetal trisomies (T21, T18, and T13) (Scotchman et al., 2020). NIPT is employed either exclusively as an initial test or confirmed by regular invasive prenatal testing. For non-invasive prenatal diagnosis of monogenic disorders, several technical and analytical obstacles still need to be resolved. Herein, we aimed to develop a new approach using cfDNA in fetal screening for AR diseases. First-trimester screening for AR diseases can be vital, specifically for populations with high numbers of consanguineous marriages. Considering these populations, a significant percentage of individuals might be carriers of one or more AR disorders, potentially inducing a high burden on families and respective communities. Currently, the only preventive measure for AR in these populations involves premarital screening or carrier screening and subsequent preimplantation genetic testing for monogenetic disorders if both partners are found to be carriers (Alfadhel et al., 2021, 2022).

Therefore, the availability of suitable screening methods for a wide range of AR disorders within a specific community is a promising strategy for early preventive measures (Alfadhel et al., 2019; Alyafee et al., 2021b). The probability of a fetus being affected or healthy has been successfully reported in previous reports; however, limitations such as maternal genetic DNA interference and/or low fetal fraction were found to persist. In the present study, we enriched the percentage of fetal cfDNA by double-size selection purification, as previously described (Liang et al., 2018; Hu et al., 2019). Subsequently, to overcome the effect of double purification of markedly low levels of cfDNA starting material, we used a WGA approach using an Ampliseq library preparation kit. This facilitated the amplification of our target cffDNA to reach detectable levels, similar to the direct detection of point mutations. To confirm the applicability of our developed approach, we attempted to detect the fetal status of four pregnant women carriers and their partners with different AR diseases. The first case was a couple who were carriers of an extremely rare and likely pathogenic mutation with a previous history of an affected child. Our approach enabled the detection of high-risk fetal aneuploidy, mosaic trisomy 21, as well as allowed the accurate identification of the fetal status of two different genes within two different chromosomes. Moreover, reducing maternal genetic interference allowed the detection of an affected homozygous fetus for *LZTR1* gene variants causing schwannomatosis disease and wild-type status for the *DVL2* gene. Case 2 presented similar findings, where we detected the wild-type status of the sickle cell disease mutation in a normal healthy fetus. In addition, we detected non-maternal alleles to rule out the likelihood of allele dropout in determining the wild-type status.

We recruited two additional patients for further confirmation. The two couples were carriers of pathogenic variants in the *MYO7A* gene (Usher syndrome) and *RNASEH2B* (Aicardi-Goutières syndrome). The results demonstrate the ability of our approach to minimize maternal DNA interference while detecting the single-point mutation status of the fetus. Furthermore, all cffDNA results were confirmed by Sanger sequencing or arrayCGH of fetal cells obtained by amniocenteses.

In the present study, we present a successful approach for isolating and enriching fetal cfDNA to detect AR diseases. The ability to detect high-risk fetuses in the first trimester of pregnancy, especially in populations with AR diseases, has great value. The mutant allele carrier status in adults is approximately 1%, and among newborns, mutant alleles account for nearly 10% of pediatric hospitalizations and ∼20% of infant mortalities. New and effective methods, such as the method presented in the present study, that can aid in rapid prenatal diagnosis are needed to prevent genetic disorders and improve the quality of life.

## 5 Conclusion

In conclusion, this feasibility study examined a novel approach for NIPT of AR disorders. In this approach, cffDNA underwent double-size selection and purification, followed by WGA for enrichment. Using this approach, we could precisely detect the fetal status considering the corresponding family gene. Therefore, this study offers solutions to reduce maternal genetic interference and low levels of cffDNA, aiding in the more accurate detection of high-risk fetuses with AR disorders. Further confirmation with additional cases involving different genes and locations is required for optimum results to evaluate the percentage of false-positive and false-negative cases. Moreover, additional studies assessing factors that may impact the final cffDNA yield and quality should be considered to further validate this new approach.

## Data Availability

The data presented in the study are deposited in the LOVD online repository: https://databases.lovd.nl/. Individual ID # 00418812, 00418890, 00418891.

## Abbreviations

AR, autosomal recessive disorder; CGH, comparative genomic hybridization; CFF DNA, cell-free fetal DNA; IRB, Institutional Review Board; KAIMRC, King Abdullah International Medical Research Centre; NGH, National Guard Hospital; NSG, next-generation sequencing; NIPT, non-invasive prenatal testing; WGA, whole-genome amplification
